# Implant primary stability depending on protocol and insertion mode — an ex vivo study

**DOI:** 10.1186/s40729-020-00245-3

**Published:** 2020-09-03

**Authors:** Henning Staedt, Peer W. Kämmerer, Elisabeth Goetze, Daniel G. E. Thiem, Bilal Al-Nawas, Diana Heimes

**Affiliations:** 1 Private Practice and Department of Prosthodontics and Materials Science, University Medical Center Rostock, Strempelstraße 13, 18057 Rostock, Germany; 2 Department of Oral- and Maxillofacial Surgery, University Medical Center Mainz, Augustusplatz 2, 55131 Mainz, Germany

**Keywords:** Dental implant primary stability, Over-dimensioned protocol, Insertion mode, Resonance frequency analysis, Insertion torque

## Abstract

**Background:**

Dental implant primary stability is thought to be a fundamental prerequisite for the long-term survival and success. The aim of this study was to analyze the influence of protocol and insertion mode on dental implant stability ex vivo. One hundred and twenty implants were inserted either manually or machine-driven into porcine mandibles by a standard or over-dimensioned protocol. Dental implant stability was measured via resonance frequency analysis (RFA), insertion torque (IT), and torque out (TO).

**Results:**

Statistically significant higher IT and TO values were seen after standard protocol insertion (*p <* 0.05), whereas manual and machine-driven insertion mode showed equivalent values.

**Conclusions:**

The over-dimensioned protocol exceeded the primary stability values recommended for immediate implant insertion; therefore, it could be recommended as well.

## Background

A reliable option for replacing teeth is the insertion of osseointegrated implants. Dental implant primary stability (DIS) has also been reported to be a fundamental prerequisite for long-term success of dental implants [1, 2], even though osseointegration has also been achieved without a certain amount of primary stability [3, 4]. Primary stability has been defined as the ability to withstand axial, lateral, and rotational loading [5] and depends on the implants’ anchorage within the bone [6]. The creation of different implant geometries and drilling protocols seems to have improved the achievement of high primary stability within the bone. The interaction between the implants’ geometric properties, combined with the surgical drilling technique indicated for the detected bone density can contribute to obtaining low amounts of compressive stress and micromotions on the surrounding bone tissue during placement [6–8].

For evaluation of in vivo primary stability, insertion torque (IT) and resonance frequency analysis (RFA) are well-established methods [2, 9]. IT is a mechanical parameter influenced by surgical procedure [10, 11], implant design [8, 10, 11], and bone quality [2, 6, 8, 10–12]. It has been defined by the Foundation for Oral Rehabilitation as the cutting resistance of the bone during implant insertion, the friction, and has been considered an indirect value of implant primary stability [13]. Values above 32 Ncm indicate that the implant is firmly embedded in the bone and mechanically stable [2, 4, 14]. RFA assumes that the frequency is directly related to the stiffness of the bone-implant interface and the surrounding bone [5, 14, 15]. High values show a stable implant and allow verification of osseointegration and secondary stability over time [11, 16]. RFA is measured by Ostell®-devices (Osstell ISQ, Osstell, Göteborg, Sweden). A sensor (SmartPeg, Osstell, Göteborg, Sweden) is mounted on the implant and is vibrated by moving it with magnetic pulses. With increasing stiffness of the bone-implant interface, the vibration frequency of the sensor increases. While RFA is expressed in hertz, implant stability quotient (ISQ) is the scale used to quantify RFA values (range 1–100) [14, 17]. Even though RFA has been reported to be a reliable, reproducible, and objective method to measure the stiffness of bone-implant-complex [11, 18], it has also been reported that RFA data from immediately placed implants could be misleading in increasing in terms of predicting primary stability [19–21]. Therefore, this value needs to be supported by another means of quantifying anchorage, especially for immediate loading protocols [18]. The reverse torque test or torque out (TO) could be used in vitro*/*ex vivo, as it gives information on the ability of the abutment or the whole implant to withstand a given torque value [10, 14, 22–25]. As this test means to unscrew the implant, this method is inappropriate for clinical use.

Patient-dependent factors affecting implant stability include bone quality and quantity. Greater stability was achieved in more dense bone [1, 2, 4, 6, 8, 10, 12, 14, 15, 18]; here, failure rates accumulated in the upper jaw [2, 12, 15]. Age, gender, smoking status, and periodontal status also affect the DIS [15]. Implant-related factors affecting DIS vary from implant macro-geometry to surface characteristics [1, 4]. The surgical technique also influences DIS [6, 12, 14], and higher insertion torques as well as under-dimensioned drilling protocols are thought to increase the percentage of initial bone-implant contact by a better fit of the implant into the bone [4, 18, 26] which in turn reduces the amount of micromotion after implant insertion [7, 8, 10, 27].

Dental implant failure is related to numerous factors such as the quality and quantity of the bone-implant interface [4, 8, 14, 18], the type of loading [1, 4, 7, 8, 12, 14, 18, 26], implant geometry [1, 4–6, 8, 12, 15, 18, 26, 28] and surface characteristics [4, 14, 28], restoration prosthesis type, and the surgeons’ experience [8]. Even though previous studies showed higher stability values for implants inserted in under-dimensioned cavities [7, 18], in the last few years research has been directed to over-dimensioned protocols (ODP). Analyzing those, a decrease in primary and an increase in secondary stability with a shorter healing period for implants became apparent [7, 29]. Kim et al*.* compared the effect of oversized drilling sockets regarding bone-to-implant contact and bone density after 4 and 8 weeks in an in vivo dog model. They used a final drill of 4.00 mm for implants with a diameter of 4 mm in the oversized group and a final drill of 2.85 mm in the control group. The analysis could show that while the initial bone-to-implant contact at 4 weeks was lower in the oversized group, it increased within the following 4 weeks up to values of 77.38% which was higher than the values in the control group (69.52%). After 8 weeks, the mean bone density was shown to be comparable between both groups. No difference could be observed regarding the healing period [30]. Within a study analyzing the differences between under-, over-dimensioned, and intermediate-sized drilling sockets in dogs after a healing period of 2 weeks, Campos et al. could show that although the undersized drilling condition led to the highest insertion torque, new bone formation was most active in the intermediate-sized group. They concluded that high insertion torque values do not necessarily result in the most favorable biologic response [31]. The purpose of this study was to examine the effect of either standard (SP) or ODP, using three different means of quantifying dental implant primary stability. To our best knowledge, only one study evaluated the effect of manual and machine-driven insertion mode so that further analysis was done to quantify the hypothesized differences [26].

The research hypotheses were that a manual insertion mode is more advantageous and that implants inserted by ODP were expected to show a decrease in dental implant primary stability, as described previously [32].

## Methods

### Bone specimens

Twenty mandibles from fresh porcine cadavers were obtained from a local slaughterhouse. The animals did not show any macroscopic signs of any pathologic bone conditions. After removal of the surrounding soft tissue, the surfaces of the bone samples were thoroughly cleaned. Each sample was checked macroscopically for irregularities and a minimum thickness of 20 mm at the place of the intended implant placement was verified. All obtained samples were immediately used.

### Surgery

In the following, a preparation wider than recommended by the company (also if narrower than the implants’ diameter) will be termed as over-dimensioned protocol (ODP).

Osteotomies were done following the manufacturer’s protocol, using sequences of varied diameter drills. One hundred and twenty implants (HiTec Tapered Self Thread implant, Hi-Tec Implants, Herzlia, Israel; 8 mm length and 3.3/3.75 diameter) were inserted either manually or machine-driven into porcine mandibles using SP or ODP. A manual insertion mode was used for implants inserted via SP (*n* = 45) or ODP (*n* = 30). A machine-driven mode was used for additional implants inserted via SP (*n* = 45). Drilling was conducted at 850 rpm and the implant was inserted at a speed of 15 rpm using a surgical motor for dental implants (Elcomed, type SA-310, W&H Dentalwerk Bürmoos GmbH, Bürmoos, Austria). Maximum insertion torque was set at 45 Ncm when implants were machine-driven, whereas manually inserted implants were drilled in by a hand ratchet at max. 45 Ncm.

### Preparation protocol for standard osteotomy (SP)

**Fig. 1:**
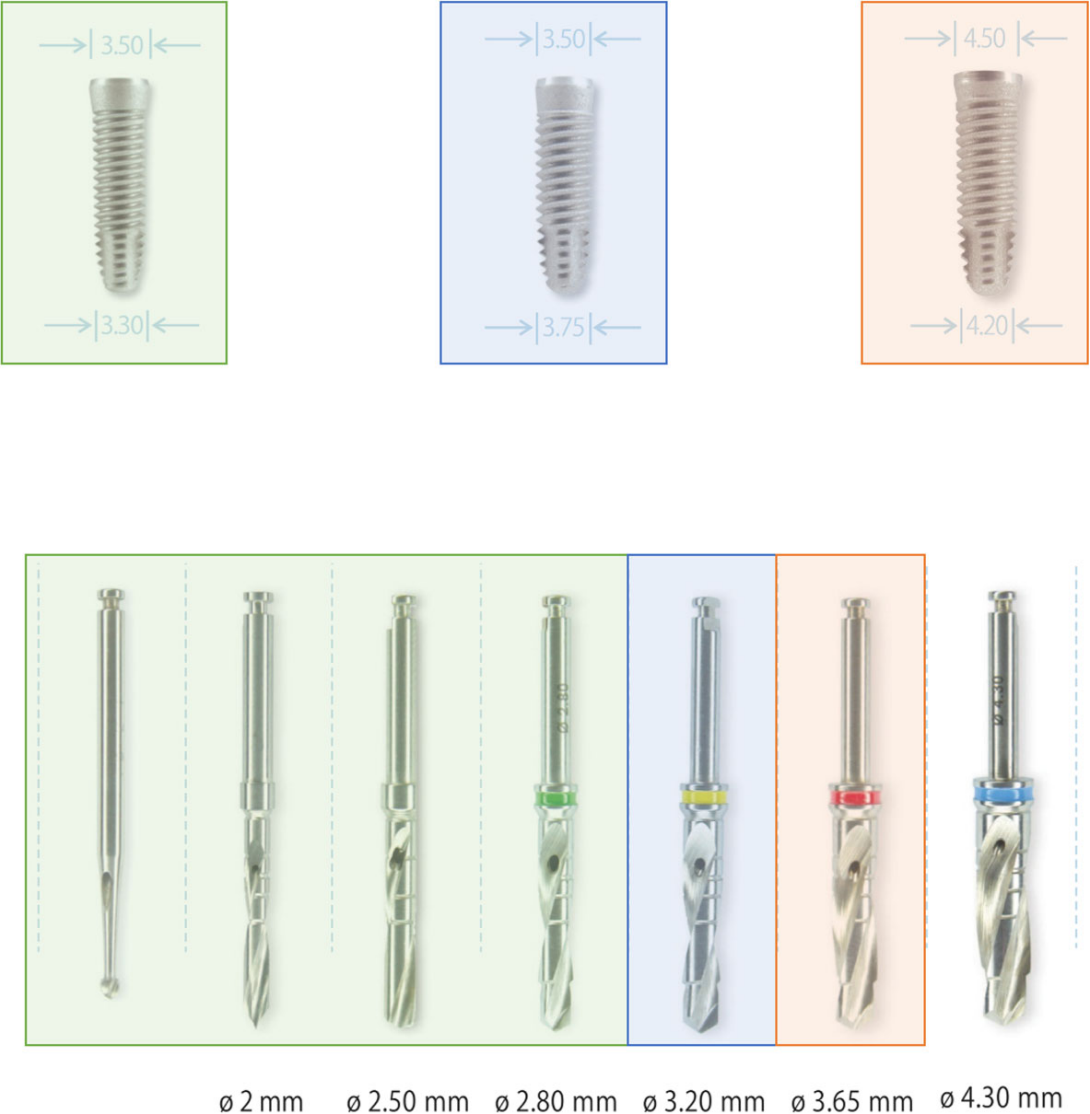
Standard protocol. This figure shows the implant types and drilling protocol used within this study. Standard protocol was conducted by a final drill of 2.80 mm for 3.3 mm implants, 3.20 mm for 3.75 mm implants, and 3.65 mm for 4.2 mm implants. Permissions for reproducing the figures were received from HI-TEC IMPLANTS LTD. Source: Product Catalogue 12th Edition [40]

Osteotomies were conducted in accordance with the manufacturer’s protocol [33]. After exposure, the bone was penetrated to a depth equivalent to the implant length using the 2-mm internal irrigation drill. Using the 2.5-mm and 2.8-mm drill, the preparation for 3.3 mm implants was completed. To achieve the space for 3.75 mm implants, the 3.2-mm drill was also used (Fig. 1).

### Preparation protocol for oversized osteotomies (ODP)

**Fig. 2:**
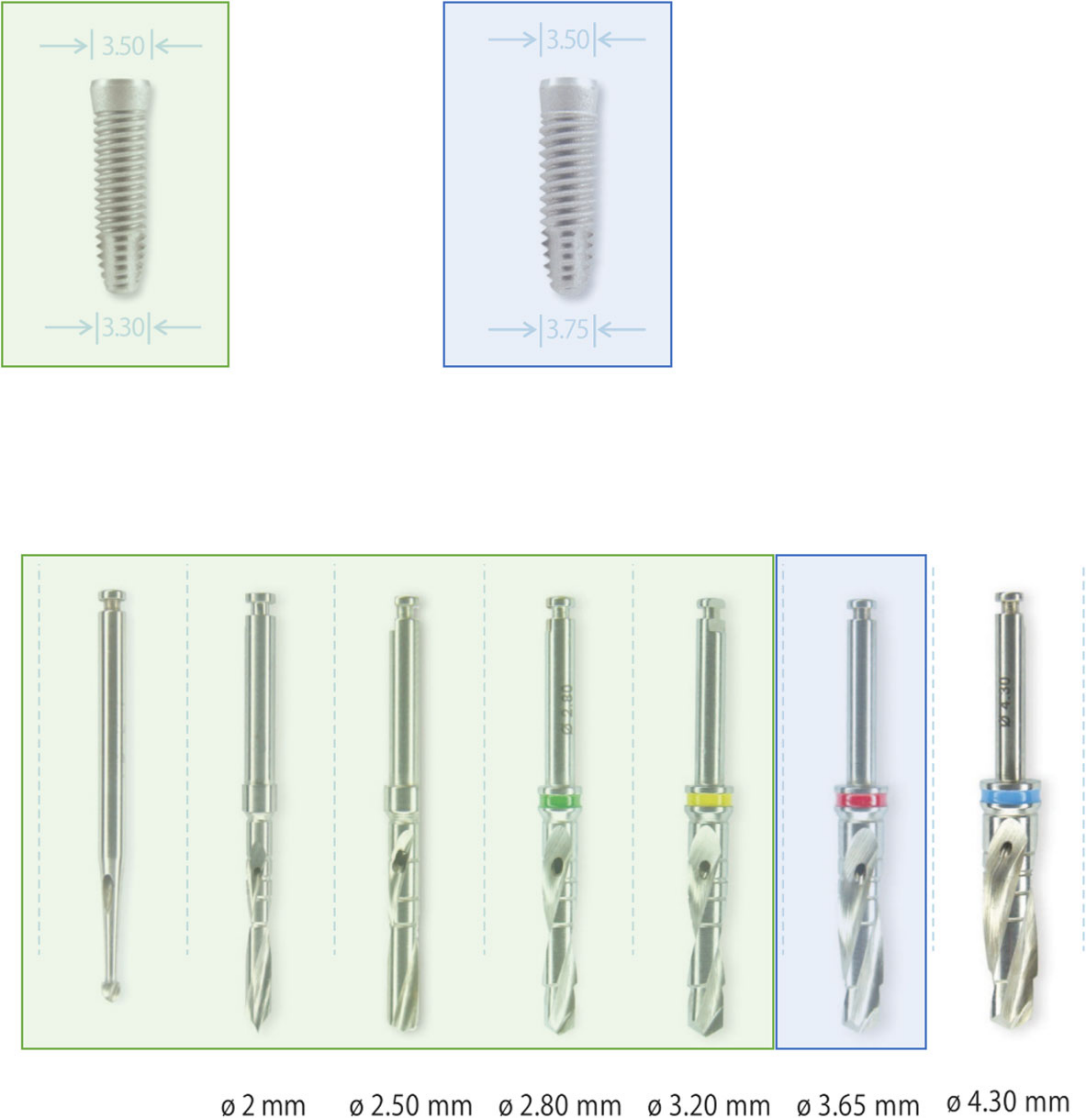
Over-dimensioned protocol. The over-dimensioned protocol was conducted by a final drill of 1 mm narrower than the implant diameter. The final drill for implants of 3.3. mm was 3.2 mm and of implants measuring 3.75 mm, it was 3.65 mm. Within this study, an over-dimensioned protocol was defined as a final drill larger than recommended by the company, which is in this case 4 or 4.5 mm wider than used in the standard protocol. Permissions for reproducing the figures were received from HI-TEC IMPLANTS LTD. Source: Product Catalogue 12th Edition [34]

This protocol repeated the steps of the standard protocol but then added a larger final drill. For the 3.3-mm implants, the final drill size was 3.2 mm; for the 3.75-mm implants, the final drill size was 3.65 mm (Fig. 2).

### RFA

To analyze the data, an Osstell® SmartPeg threaded transducer (implant diameter 3.3 and 3.75 mm: SmartPeg Type 32 with a platform of 3.5 mm, Art-Nr. 100440; implant diameter 4.2 mm: SmartPeg Type 27 with a platform of 4.5 mm, Art-Nr. 10043; Osstell, Göteborg, Sweden) was screwed onto the implant fixture with the aid of a transporter. RFA was measured by Osstell® electronic analyzer (Osstell ISQ), positioned at a distance of 3 mm from the abutment. The recorded frequency was automatically converted into implant stability quotient (ISQ) values with a range of 0 to 100 (minimum to maximum stability). The mean ISQ values were calculated.

### Torque in and torque out

Insertion torque and torque out were measured by using a customized handheld torque screwdriver with a digital output device (Mecmesin, Schwenningen, Germany) as described by Bolm et al. [35]. Torque values were recorded in Newton centimeter.

### Statistics

To analyze the differences between the measured value normality and homogeneity of variance tests (Levene statistic) were performed at first in order to check the conditions for the subsequent analysis. *p* values were obtained with either independent-samples *t* test or one-way ANOVA. In case of inhomogeneity of variance, a Welch-ANOVA was used instead. To obtain *p* values, post hoc multiple comparison Tukey test (equal variances assumed) or Games-Howell (equal variances not assumed) was used. The effect size was calculated as described in “Statistical power analysis for the behavioral sciences” by Jacob Cohen in 1988 [36]. Values are displayed as Cohen’s *d* and effect size (*r*). The statistical analyses were performed using SPSS version 24 for Windows (IBM, Armonk, New York). A *p* value *≤* 0.05 was termed significant. Values are displayed as mean plus standard deviation.

## Results

### Drilling protocol: standard versus over-dimensioned

**Fig. 3:**
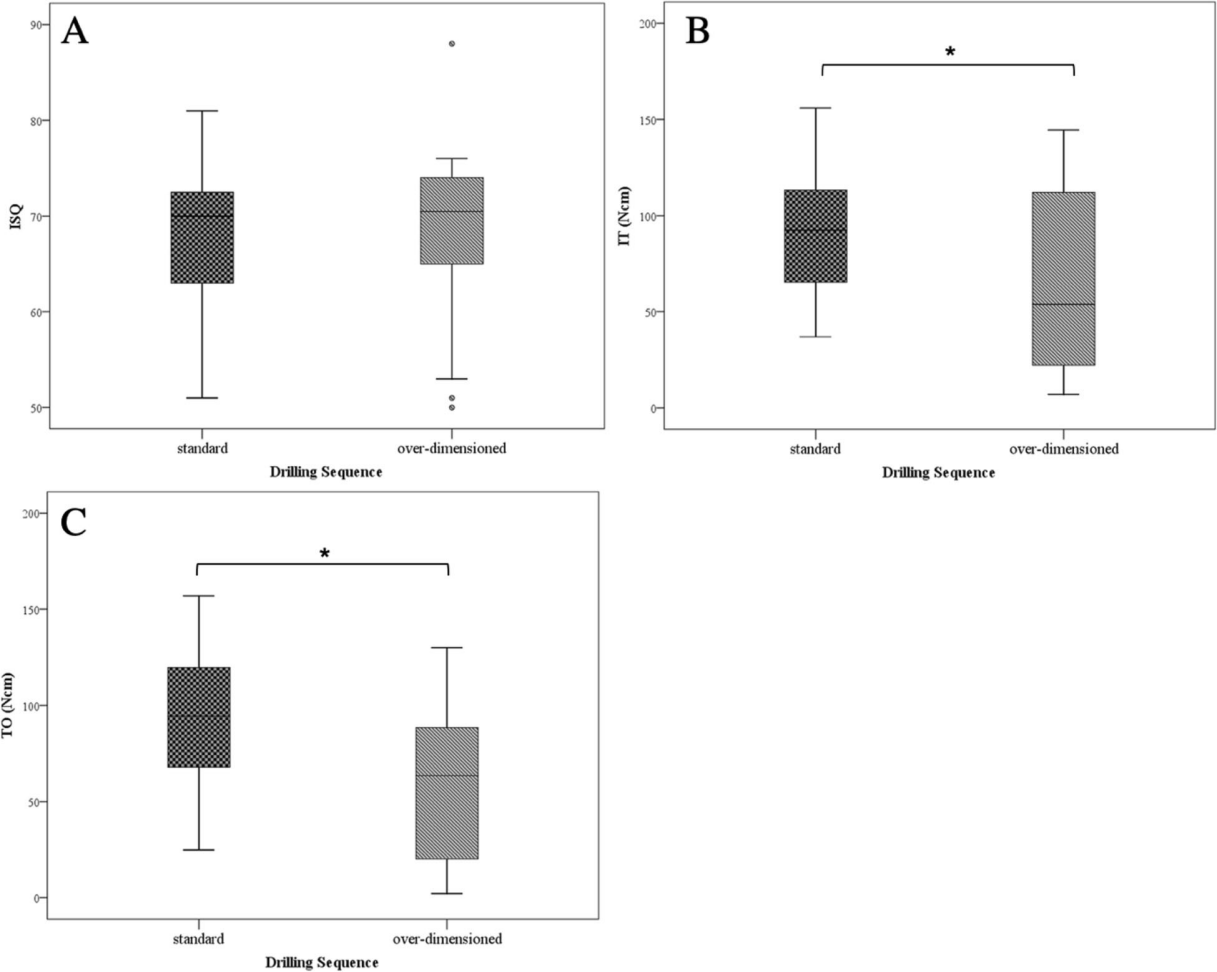
Comparison of standard and over-dimensioned protocol. The figure displayed shows the comparison between standard and over-dimensioned protocol. **a** Displays the measurements obtained by RFA. The unit is ISQ with a range of 0 to 100 (minimum to maximum stability). **b** Displays the results obtained by the torque in and **c** by the torque out test. Although, there was no statistically significant difference in ISQ between the groups, torque in and torque out tests showed significantly lower values in the over-dimensioned group compared to implant inserted via standard protocol. ISQ, implant stability quotient; IT, insertion torque; TO, torque out

No statistically significant difference in RFA could be measured (Cohen’s *d* = − 0.022, effect size *r* = 0.011, *p* = 0.260), whereas IT values were significantly higher in implants inserted via SP (90.56 ± 31.27 Ncm) in comparison with the ODP (63.74 ± 48.61 Ncm, *p =* 0.002; Cohen’s *d* = 0.656, effect size *r* = 0.312). The analyzed TO values showed similar results with higher values in the SP (93.59 ± 32.3 Ncm) compared with the ODP (58.35 ± 40.43 Ncm, *p* = 0.043; Cohen’s *d* = 0.963, effect size *r* = 0.434) (see Table 1 and Fig. 3).

**Table 1:** Comparison of standard and over-dimensioned protocol

Drilling sequence — standard protocol				Drilling sequence — over-dimensioned protocol		
	***n***	Mean (SD)	CI	***n***	Mean (SD)	CI
**ISQ**	45	68.33 (6.83)	66.14–70.51	30	68.5 (8.82)	65.08–71.92
**IT (Ncm)**	45	90.56 (31.27)	80.56–100.56	30	63.74 (48.61)	44.89–82.59
**TO (Ncm)**	45	93.59 (32.3)	83.27–103.92	30	58.35 (40.43)	42.67–74.02

*ISQ* implant stability quotient, *IT* insertion torque, *TO* torque out, *SD* standard deviation, *CI* confidence interval

### Insertion mode: manual versus machine-driven, standard protocol

**Fig. 4:**
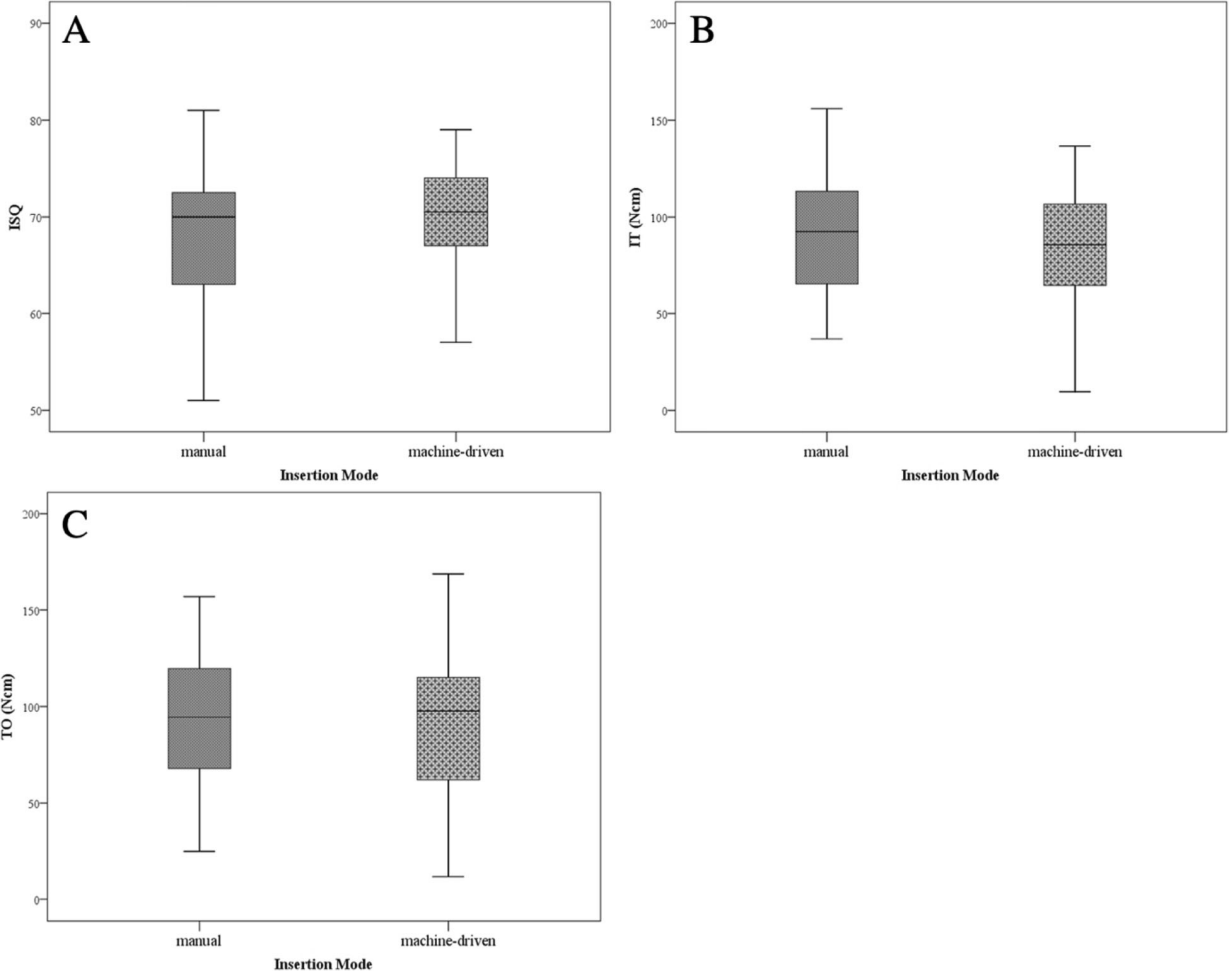
Comparison of manual and machine-driven insertion mode. This figure displays the comparison between manual and machine-driven insertion mode. **a** Displays the results obtained by the RFA test, indicated as ISQ (0–100). **b** Displays the measurements obtained by the torque in and **c** by the torque out test. There was no statistically significant difference between the groups. ISQ, implant stability quotient; IT, insertion torque; TO, torque out

No statistically significant difference of RFA (Cohen’s *d* = –0.309, effect size *r* = –0.153, *p* = 0.185), IT (Cohen’s *d* = 0.21, effect size *r* = 0.104, *p* = 0.937), and TO (Cohen’s *d* = 0.109, effect size *r* = 0.054, *p* = 0.490) could be shown between manual and machine-driven insertion mode (see Table 2 and Fig. 4).

**Table 2:** Comparison of manual and machine-driven insertion mode

	Insertion mode — manual insertion			Insertion mode — machine-driven insertion		
	***n***	Mean (SD)	CI	***n***	Mean (SD)	CI
**ISQ**	45	68.33 (6.83)	66.14–70.51	45	70.25 (5.52)	68.38–72.12
**IT (Ncm)**	45	90.56 (31.27)	80.56–100.56	45	83.94 (31.81)	73.17–94.7
**TO (Ncm)**	45	93.59 (32.3)	83.27–103.92	45	89.80 (37.32)	77.18–102.43

*ISQ* implant stability quotient, *IT* insertion torque, *TO* torque out, *SD* standard deviation, *CI* confidence interval

### Implant geometry: standard protocol

No statistically significant difference regarding ISQ (*p* = 0.353), IT (*p* = 0.099), or TO (*p* = 0.337) could be measured between implants of different diameter. Neither was there a statistically significant difference between implants of different lengths (ISQ: *p* = 0.164, IT: *p* = 0.303, TO: *p* = 0.246) (see Tables 3 and 4).

**Table 3:** Comparison of implant diameter

	3.3 mm		3.75 mm		4.2 mm	
	Mean (SD)	CI	Mean (SD)	CI	Mean (SD)	CI
**ISQ**	66.33 (4.59)	63.79–68.88	69.00 (5.98)	64.72–73.28	69.87 (8.88)	64.94–74.78
**IT (Ncm)**	102.65 (28.42)	86.91–118.39	90.97 (27.54)	71.27–110.67	78.19 (33.28)	59.76–96.62
**TO (Ncm)**	94.54 (29.09)	78.43–110.65	81.28 (28.89)	60.67–101.88	100.86 (36.89)	80.43–121.29

*ISQ* implant stability quotient, *IT* insertion torque, *TO* torque out, *SD* standard deviation, *CI* confidence interval

**Table 4:** Comparison of implant length

	8.0 mm		10.0 mm		11.5 mm		13.0 mm		16.0 mm	
	Mean (SD)	CI	Mean (SD)	CI	Mean (SD)	CI	Mean (SD)	CI	Mean (SD)	CI
**ISQ**	65.5 (8.40)	58.48–72.52	73.17 (3.60)	69.39–76.95	67.11 (6.09)	62.43–71.79	66.15 (8.15)	59.43–73.07	70.67 (4.97)	66.84–74.49
**IT (Ncm)**	98.23 (18.56)	82.71–113.74	99.49 (43.73)	53.60–145.48	101.02 (36.80)	72.74–129.31	73.79 (23.57)	54.08–93.50	82.22 (28.77)	60.11–104.34
**TO (Ncm)**	102.26 (31.77)	75.70–128.83	103.08 (43.25)	57.69–148.48	103.98 (35.35)	76.81–131.15	73.65 (26.56)	51.44–95.85	86.90 (21.32)	70.51–103.29

*ISQ* implant stability quotient, *IT* insertion torque, *TO* torque out, *SD* standard deviation, *CI* confidence interval

## Discussion

This study was performed in order to investigate changes in primary stability within an experimental setup of different insertion protocols and insertion modes. In order to obtain a high level of diagnostic certainty, three different methods for measurement of primary stability were recorded. As a secondary outcome parameter, potential differences between implants of different length and diameter have been evaluated. Within this study, no statistically significant difference could be shown so that the groups were not separated regarding the implant’s geometry while further analyzing the insertion mode and the drilling protocol. In line with the research hypothesis, the ODP showed significant lower stability values when compared with implants inserted via SP. The optimization of loading protocols requires establishing a patient-specific protocol [14]. The relationship between shape and shear strength during implant placement modulates the bone compression [11]. Osteotomy techniques with a narrower final drill have been shown to create a better bone-to-implant contact with enhanced primary stability values [18]. As micromotions’ risk osseointegration, there is consensus that implant stability immediately and early after placement is desirable [7]. Primary stability is generally associated with the expectation of good secondary stability, which would ensure the likelihood of implant success and osseointegration under-dimensioned protocols are thought to induce a tight contact immediately after placement: in soft bone, the use of wide diameter implants and an under-dimensioned preparation has been recommended to preserve the cortical layer [4, 18]. A study by Campos et al. reported a significant increase in bone-to-implant contact between SP and under-dimensioned protocols, but they could not show any effect in histometrical parameters [7]. Okazaki et al. analyzed the impact of different loading protocols on implant removal torque values and showed stability values to be up to eleven times lower at the time of insertion in ODPs, whereas no difference between SP and ODP could be observed after a healing phase of 6 to 12 weeks [10]. However, caution is recommended when using under-dimensioned drilling protocols: although high insertion torques ensure a greater initial implant stability and prevent adverse micromotions under loading, the induced over-compression could jeopardize the healing process [10, 28]. In addition, high stress is known to alter angiogenesis and impair new vessel formations, to induce local hypoxia and necrosis, inhibiting new bone formation and to adversely affect implant stability [4, 7, 10, 28, 37]. Also, higher degrees of primary stability do not necessarily translate into high degrees of secondary stability [11]. After a latency of 1 week, necrotic bone fills the space between bone and implant whereupon a bone remodeling takes place within 1 to 3 weeks [11, 37]. By using larger drilling dimensions, the bone-implant interface is filled with a blood clot so intermembranous-like ossification takes place without a formation of necrotic bone spots [7]. With a shorter healing period of two instead of 4 weeks, this “healing chamber” is considered a key factor for secondary stability. Due to its different healing patterns, it does not have to undergo tissue remodeling [11, 38, 39]. Contrary to this, ODPs could lead to displacements above 50–150 μm resulting in fibrous bone formation and a poor long-term stability [14]. With a reduction in primary stability and a secondary stability similar to SPs [10], test results have shown a shorter healing period [11, 38, 39] with lower degrees of compressive stress transferred to the surrounding bone. Nevertheless, in this study, implants inserted via ODP exceeded the clinically established lower limits for primary insertion. A minimum of 45 Ncm in IT and 70 units in ISQ was reported to be required to obtain the necessary stability for primary insertion [40]. Therefore, based on the results at hand, the use of ODPs might be preferable to reduce the level of compressive stress, especially in patients with dense bone.

Contrary to the research hypothesis, there was no difference in primary stability between manually and machine-driven inserted implants. To date, little is known about the influence of the insertion mode on the dental implant primary stability. Novsak et al. assumed a better primary stability in implants inserted manually and suspected that this behavior was related to a higher tactile sensation in hand-driven implantation [26]. In contrast, movements during insertion and a greater amount of rotational stress due to higher torque values could explain a poor outcome during manual insertion. In accordance with the results of the present study, it can be assumed that there is no difference between manual and machine-driven insertion modes and that surgical experience in general and especially in using either procedure seems to be more important than the insertion mode itself.

## Conclusions

The aim of this study was to analyze the influence of protocol and insertion mode on dental implant primary stability ex vivo. Implants inserted via standard protocol showed higher stability values than implants inserted via an over-dimensioned protocol. Interestingly, the study demonstrated that the latter nevertheless exceeded the primary stability recommended for immediate implant insertion so that the use of ODPs might be preferable to reduce the level of compressive stress. This study also showed that manual and machine-driven insertion modes exhibited equivalent primary stability values. Under the limitations of this study, it can be hypothesized that the choice of implant type in combination with the surgical drilling technique adapted to the quality of the bony implant site [1, 2, 4, 6–8] as well as patient-related factors [15] and the surgical experience [8] seems to be more important than the insertion mode itself. In the absence of guidelines and in consideration of the high number of implants inserted each year, further studies are needed to confirm or disprove these results and to improve the knowledge within this area.

## Data Availability

The dataset supporting the conclusions of the article is included within the article and its additional files. The raw data analyzed during the current study is available from the corresponding author on reasonable request.

## Abbreviations

DIS: Dental implant primary stability
ISQ: Implant stability quotient
IT: Insertion torque
ODP: Over-dimensioned protocol
RFA: Resonance frequency analysis
SP: Standard protocol
TO: Torque out
